# SOCIAL CHANGE, SOCIAL INSTITUTIONS, AND COHORTS: CONTEXTUALIZING MEN'S HEALTH IN LATER LIFE

**DOI:** 10.1093/geroni/igz038.780

**Published:** 2019-11-08

**Authors:** Jessica Kelley

**Affiliations:** Case Western Reserve University, Cleveland, Ohio, United States

## Abstract

Research links men’s health to their participation in, and access to, social institutions such as marriage, education, and work. However, these institutions have undergone significant social change in the past century, altering their scope and influence on men’s health. We tie together several important concepts from sociology and gerontology to provide an explanatory framework for older men’s differential health profiles within and between cohorts, and over time. First, we address the gendered life course which are the structural and social arrangements that create distinctive experiences over the life course for men and women. Second, we employ the concept of cohort analysis to capture social changes in the institutionalized life course. Finally, we utilize the cumulative dis/advantage framework to help understand within-cohort differentiation in health status as men age. Taken together, we can better understand health, longevity, and disability profiles for older men and how these have shifted over time.

